# Mortality in Frankfurt am Main, Germany, 2020–2023: higher excess mortality during an influenza wave in 2022 than during all COVID-19 waves altogether

**DOI:** 10.3205/dgkh000533

**Published:** 2025-03-04

**Authors:** Ursel Heudorf, Bernd Kowall

**Affiliations:** 1 Institute of Hygiene and Environmental Medicine, Justus Liebig University, Gießen, Germany; 2 Institute for Medical Informatics, Biometry and Epidemiology, University Hospital, Essen, Germany

**Keywords:** SARS-CoV-2, COVID-19, pandemic, pandemic waves, mortality, influenza, heat

## Abstract

**Introduction:**

Mortality during the SARS-CoV-2 pandemic was studied in many countries. The results were strongly influenced by the chosen calculation method, the adjustment to the ageing of the population and the reference periods used. Smaller-scale studies sometimes showed considerable differences within countries, but it is unclear whether the differences within a country were due to the fact that the studies were small (sampling error) or whether they were true differences. In an earlier small-scale study in Frankfurt, we examined mortality during the first two years of the pandemic. Our aim was to continue this analysis until the end of 2023, for the first time taking into account other factors influencing mortality such as influenza and heat.

**Method:**

We obtained population data for Frankfurt am Main for 2016–2023 from the Municipal Office of Statistics, City of Frankfurt/Main, mortality data from 2016 to 2023 from the Hessian State Office for Health and Care, data on SARS-CoV-2 and influenza notifications from the homepage of the Robert Koch-Institute and weather data from the homepage of the German Meteorological Office. For calculating standardized mortality ratios (SMR= observed number of deaths divided by the expected number of deaths), we multiplied the mean mortality rate for 5 age groups from 2016–2019 with the total numer of residents in those age groups in the further years or periods, and finally added the numbers of expected deaths per age group.

**Results:**

The update of the assessment of mortality adjusted for age and population trend in the years 2020–2023 in Frankfurt am Main shows an excess mortality (SMR 1.029; 95% CI 1.004–1.054, +185 excess deaths) in 2022, followed by a negative excess mortality in 2023 (SMR 0.972; 95% CI 0.948–0.996). In the years 2020 and 2021 however, no increase in excess mortality had been found (2020: SMR 0.976; 95% CI 0.951–1.001; 2021: 0.998; 95% CI 0,973–1.023). In the second wave of the SARS CoV-2 pandemic with the Wuhan type (fall 2020), a significantly increased mortality was found (SMR 1.106; 95% CI 1.066–1.147, +274 deaths), as well as during the first four waves overall (Wuhan, Alpha and Delta type) (SMR 1.023; 95 CI 1.001–1.045), whereas no increased mortality occurred during the further waves with the Omikron variant in 2022 (SMR 0.988; 95% CI 0.963–1.014). The increased mortality in 2022 was associated with an influenza wave in the last 6 weeks of the year, which had led to a strong increase in mortality (SMR 1.250; 95% CI 1.170–1.330).

**Discussion:**

During the SARS-CoV-2 pandemic, significant excess mortality occurred in Frankfurt am Main only in the second wave at the end of 2020 before vaccination was introduced; in all other waves, no significant excess mortality was recorded. Overall, there was a non-significant negative excess mortality in Frankfurt am Main in 2020 and 2021 and a significant negative excess mortality in 2023. In 2022, however, a significant excess mortality was observed, which could not be attributed to SARS-CoV-2 but to a short, intense wave of influenza in the last 6 weeks at the end of that year, which had also led to a significant increase in mortality throughout Germany. This influenza wave was associated with an excess mortality rate in Frankfurt am Main, which was higher than in any wave of the SARS-CoV-2 pandemic in Frankfurt am Main. The number of excess deaths during that influenza waves was larger than the excess deaths during all SARS-CoV-2 waves altogether. This remarkable fact should be taken into account when dealing with the evaluation of the pandemic, a process which is increasingly beeing called for in many ways in Germany but is still pending.

## Zusammenfassung

**Einleitung:**

Die Sterblichkeit während der SARS-CoV-2-Pandemie wurde in vielen Ländern untersucht. Die Ergebnisse waren stark von der gewählten Berechnungsmethode, der Anpassung an die Alterung der Bevölkerung und den verwendeten Referenzzeiträumen beeinflusst. Kleinräumige Studien zeigten zum Teil erhebliche Unterschiede innerhalb der Länder, aber es ist unklar, ob diese Unterschiede innerhalb eines Landes auf die Tatsache zurückzuführen sind, dass die Studien klein waren (Stichprobenfehler), oder ob es sich um echte Unterschiede handelt. In einer früheren kleinräumigen Studie in Frankfurt untersuchten wir die Mortalität während der ersten beiden Jahre der Pandemie. Unser Ziel war es, diese Analyse bis Ende 2023 fortzusetzen und dabei erstmals auch andere Einflussfaktoren auf die Sterblichkeit, wie Influenza und Hitze, mit einzubeziehen.

**Methode:**

Wir bezogen Bevölkerungsdaten für Frankfurt am Main für die Jahre 2016 bis 2023 vom Statistischen Amt der Stadt Frankfurt am Main, Mortalitätsdaten von 2016 bis 2023 vom Hessischen Landesamt für Gesundheit und Pflege, Daten zu SARS-CoV-2 und Influenza-Meldungen von der Homepage des Robert Koch-Instituts und Wetterdaten von der Homepage des Deutschen Wetterdienstes. Zur Berechnung der standardisierten Sterblichkeitsraten (SMR= beobachtete Zahl der Todesfälle geteilt durch die erwartete Zahl der Todesfälle) multiplizierten wir die mittlere Sterblichkeitsrate für 5 Altersgruppen von 2016–2019 mit der Gesamtzahl der Einwohner in diesen Altersgruppen in den weiteren Jahren oder Zeiträumen und addierten schließlich die Zahl der erwarteten Todesfälle pro Altersgruppe.

**Ergebnisse:**

Die Aktualisierung der alters- und bevölkerungsadjustierten Sterblichkeit für die Jahre 2020-2023 in Frankfurt am Main zeigt eine Übersterblichkeit (1,029, 95% Konfidenzintervall KI 1,004–1,054, +185 Übersterbefälle) im Jahr 2022, gefolgt von einer negativen Übersterblichkeit im Jahr 2023 (SMR 0,972; 95% KI 0,948–0,996). In den früheren Jahren 2020 und 2021 wurde hingegen kein Anstieg der Übersterblichkeit festgestellt (2020: SMR 0,976; 95% CI 0,951–1,001; 2021: 0,998; 95% CI 0,973–1,023). In der zweiten Welle der SARS-CoV-2-Pandemie mit dem Wuhan-Typ (Herbst 2020) wurde eine erhöhte Sterblichkeit festgestellt (SMR 1,106; 95% CI 1,066–1,147, +274 Todesfälle), ebenso wie während der ersten vier Wellen insgesamt (Wuhan-, Alpha- und Delta-Typ) (SMR 1,023; 95 KI 1,001–1,045), während in den weiteren Wellen mit der Omikron-Variante im Jahr 2022 keine erhöhte Sterblichkeit auftrat (SMR 0,988; 95 KI 0,963–1,014). Die erhöhte Sterblichkeit im Jahr 2022 stand im Zusammenhang mit einer Influenzawelle in den letzten 6 Wochen des Jahres, die zu einem signifikanten Anstieg der Sterblichkeit geführt hatte (SMR 1,250; 95% KI 1,170–1,330).

**Diskussion:**

Während der SARS-CoV-2-Pandemie trat eine signifikante Übersterblichkeit in Frankfurt am Main nur in der zweiten Welle Ende 2020 auf, bevor die Impfung eingeführt wurde; in allen anderen Wellen wurde keine signifikante Übersterblichkeit verzeichnet. Insgesamt gab es in Frankfurt am Main eine negative Übersterblichkeit in den Jahren 2020 und 2021 und im Jahr 2023. Im Jahr 2022 war jedoch eine signifikante Übersterblichkeit zu beobachten, die nicht auf SARS-CoV-2 zurückzuführen war, sondern auf eine kurze, heftige Influenzawelle in den letzten 6 Wochen am Ende des Jahres, die auch deutschlandweit zu einem deutlichen Anstieg der Sterblichkeit geführt hatte. Diese Grippewelle war in Frankfurt am Main mit einer Übersterblichkeitsrate verbunden, die höher war als in jeder Welle der SARS-CoV-2-Pandemie in Frankfurt am Main, wobei die Zahl der Exzesstoten während der Influenzawelle die aller SARS-CoV-2-Wellen zusammen überstieg. Diese bemerkenswerte Tatsache sollte bei der in Deutschland vielfach geforderten, aber noch ausstehenden Aufarbeitung der Pandemie berücksichtigt werden.

## Introduction

During the COVID-19 pandemic, many measures were taken worldwide to prevent infections and severe cases, especially deaths. In the first weeks of the pandemic, it soon became already apparent that elderly people with pre-existing conditions, especially residents of care facilities for the elderly, were at a particularly high risk of becoming seriously ill with SARS-CoV-2 and dying from it. Evaluations of the first two years of the pandemic showed very different levels of excess mortality in different countries [1]. Due to the uncertainties in the information on the death certificates or the incomplete notifications under the German Infection Protection Act, calculation of overall mortality was generally preferred to the calculation of mortality from COVID-19 [2], [3].

In addition, the results on excess mortality (total mortality) were found to be highly dependent on the calculation methods used, the adjustment to population development, as well as to the age development of the population [1], [4], [5], [6]. Depending on the calculation method, an excess mortality of 88,446 to 203,000 was calculated for Germany in the years 2020 to 2021, for example [1]. Levitt et al. [1] calculated an excess mortality of 54,740 for Germany with age adjustment, whereas without age adjustment the value was more than twice as high at 128,557. Differently selected comparison periods – with otherwise identical calculation methods – also lead to very different results [5], [7]; however, the ranking of countries in international comparison hardly depended on the calculation method [7]. 

In the United States of America, a cross-sectional study using state-level mortality 2020–2022 data exhibited a positive association of stringent COVID-19 restriction with a reduced pandemic mortality [8]. However, in the large-scale C-MOR project encompassing 24 countries worldwide, the stringency index of control measures was both positively and negatively associated with excess mortality during 2020 and 2021 [9].

Comparisons between countries with different vulnerability – defined by the level of gross domestic product, income inequality and the proportion of the population below the poverty line – show that mortality rates were still largely comparable in the first wave of the pandemic. But at the end of 2020 mortality rose significantly in all countries, particularly in the vulnerable countries with a low gross domestic product, high income disparity and a higher proportion of the population below the poverty line. This excess mortality largely persisted until after the end of the pandemic in the vulnerabe countries, whereas in most of the less vulnerable countries it was followed by a negative excess-mortality resulting in only a small, non-significant cumulative excess mortality or even a cumulative negative excess-mortality (e.g. Denmark –3.0%, Sweden –3.5% and New Zealand –3.6%) [10]. In addition, very large differences in mortality, here age standardized years of life lost, were observed in the regions within individual countries [11]. This suggests that a closer look should be taken at smaller-scale settings. 

In our previous small-scale study we did not detect any significant excess mortality (–2.4% and –0.2%) in Frankfurt am Main, Germany, during the years 2020 and 2021 after adjusting for the age and population trend. In the second pandemic wave in autumn 2020 even before the start of vaccinations, a significant excess mortality (+10.6%) was found. This was not the case in the other waves 1, 3 and 4 with wild type, and Alpha- and Delta variant being predominant [12]. The aim of this study was to analyze and discuss mortality in Frankfurt in 2022 and 2023, with extremely high notification rates für SARS-CoV-2 in 2022. For the first time, the risks from SARS-CoV-2 but also from other factors influencing mortality such as influenza and heat will be considered.

## Methods

Population data for Frankfurt am Main for 2016–2021, referring to the population for 31^st^ December were taken from the data published in annual reports of each year of the Municipal Office of Statistics, City of Frankfurt am Main and online [13]. For our further calculations we used midyear populations, obtained as the average of the open-source population figures of two consecutive years for 31^st^ December. For the years 2022 and 2023, we used the population data as of June 30^th^, provided upon request by the Municipal Office of Statistics.

The data on death cases, compiled by the Hessian Statistical Office (HSL), were provided by the Hessian State Office for Health and Care [14]. 

Data on notification of SARS-CoV-2 and influenza infections were taken from the RKI (Robert Koch Institute) database [15]. They are available as cases per reporting week and year for different age groups (5-year intervals: 0–4y, 5–9y, 10–14y … +80y). 

The definitions of the different waves in Germany were obtained from Tolksdorf et al. [16]: wave 1 calendar week (CW) 10 to 20/2020; wave 2 CW 40/2020 to 8/2021; wave 3 CW 9 to 23/2021; wave 4 CW 31 to 51/2021, wave 5 CW 52/2021–21/2022, wave 6 CW starting in CW 22 [16]. Because further definitions have not yet been published by the Robert Koch-Institute, we defined the duration of waves 6 and 7 according to the SARS-CoV-2 notification rates in Frankfurt: wave 6: CW 22–33/2022 and wave 7 CW 34–46/2022.

Data on daily mean temperatures recorded at the weather station in Frankfurt am Main were taken from the homepage of the German National Meteorological Service [17].

For the summer months, June–August, weekly mean temperatures (Monday–Sunday) were calculated. According to Winklmayr et al. [18] and Winklmayr and an der Heiden [19] weeks with mean weekly temperatures above a threshhold – here 20°C – were defined as heat weeks. 

As described in our previous publication [12] we estimated age-adjusted standardized mortality ratios SMR by firstly calculating mortality rates for 2016–2019 separately for five age groups (0–29, 30–59, 60–69, 70–79, ≥80 years) for each year. Each age-specific mean mortality rate was multiplied by the population of the corresponding age group in each of the following years 2020 to 2023 to give the expected number of deaths in those years for each age group. The total number of expected deaths for each of the years 2020 to 2023 was obtained by adding the expected numbers of deaths for the five age groups. This total number of expected deaths was used as the denominator of SMR, whereas the observed numbers of deaths were used as the numerator. This procedure was applied not only to the complete years but also to all calendar weeks from 2000 to 2023, to assess yearly and weekly excess mortality – as differences (observed–expected deaths and SMR (observed/expected deaths) The same procedure was applied for heat weeks in 2020–2023. Additionally, we repeated the calculation for annual excess mortality in 2020–2023 by differentiating into 8 age groups (0–29, 30–59, 60–64, 65–69, 70–74, 75–79, 80–84, and ≥85 years). 

Finally, we compiled population data, the observed deaths and weather data from January 1^st^ 2000 to December 31^st^ 2023. We calculated weekly death rates per 100,000 population and compared these data with weekly notification rates per 100,000 population for SARS-CoV-2 and influenza, and with heat weeks. 

## Results

The follow-up of the age trend-adjusted excess mortality analysis in Frankfurt am Main during the pandemic years up to 2023 is shown in Table 1 (Tab. 1). While negative excess mortality SMRs (0.976; 95% CI 0.951–1.001 and 0.998; 95% CI 0.973–1.023) were found in 2020 and 2021, a positive SMR 1.029 (95% CI 1.004–1.054) was found in 2022 and a slighty negative SMR 0.972 (95% CI 0.948–0.996) was found again in 2023. The data of the respective calculation for 8 age groups is shown in Attachment 1 resulting in higher SMRs and excess deaths.

**Figure 1:**
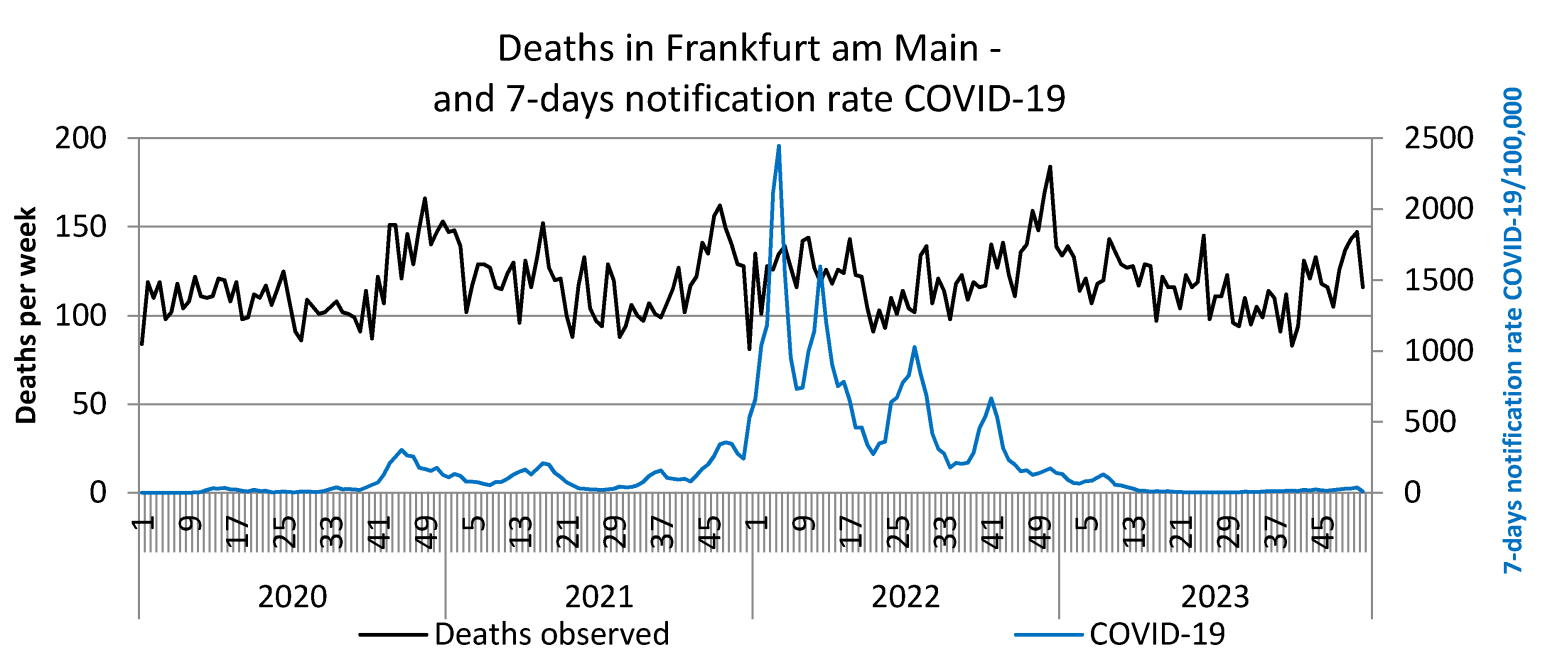
Weekly deaths (unadjusted) and notification rates COVID-19/100,000 in Frankfurt am Main, Germany

**Figure 2:**
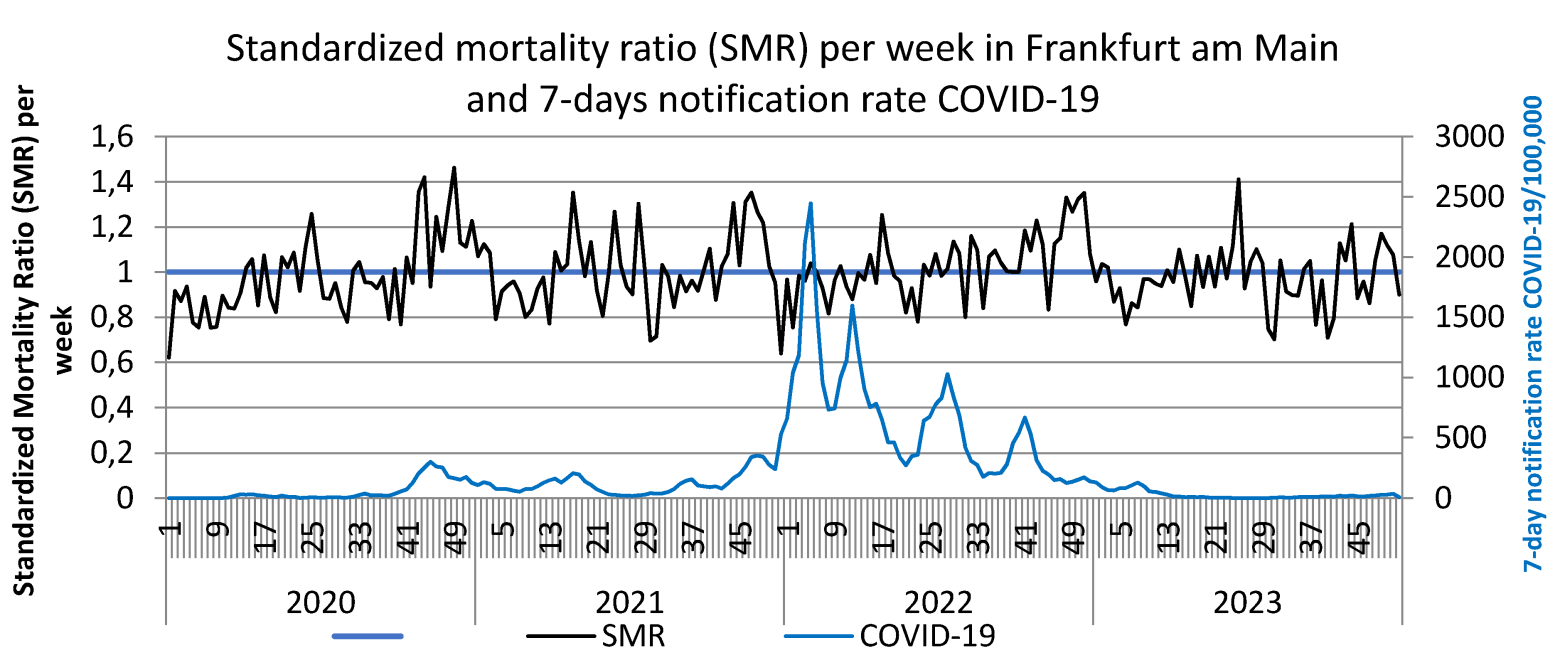
Weekly standardized mortality ratio (SMR) and 7-day notification rates COVID-19/100,000 in Frankfurt am Main, Germany

Furthermore, the 7-day SARS-CoV-2 notification rates from January 2020 to December 2023 are plotted against the observed deaths per week (Figure 1 (Fig. 1)) and against the standardized mortality ratio SMR (Figure 2 (Fig. 2)). 

**Figure 3:**
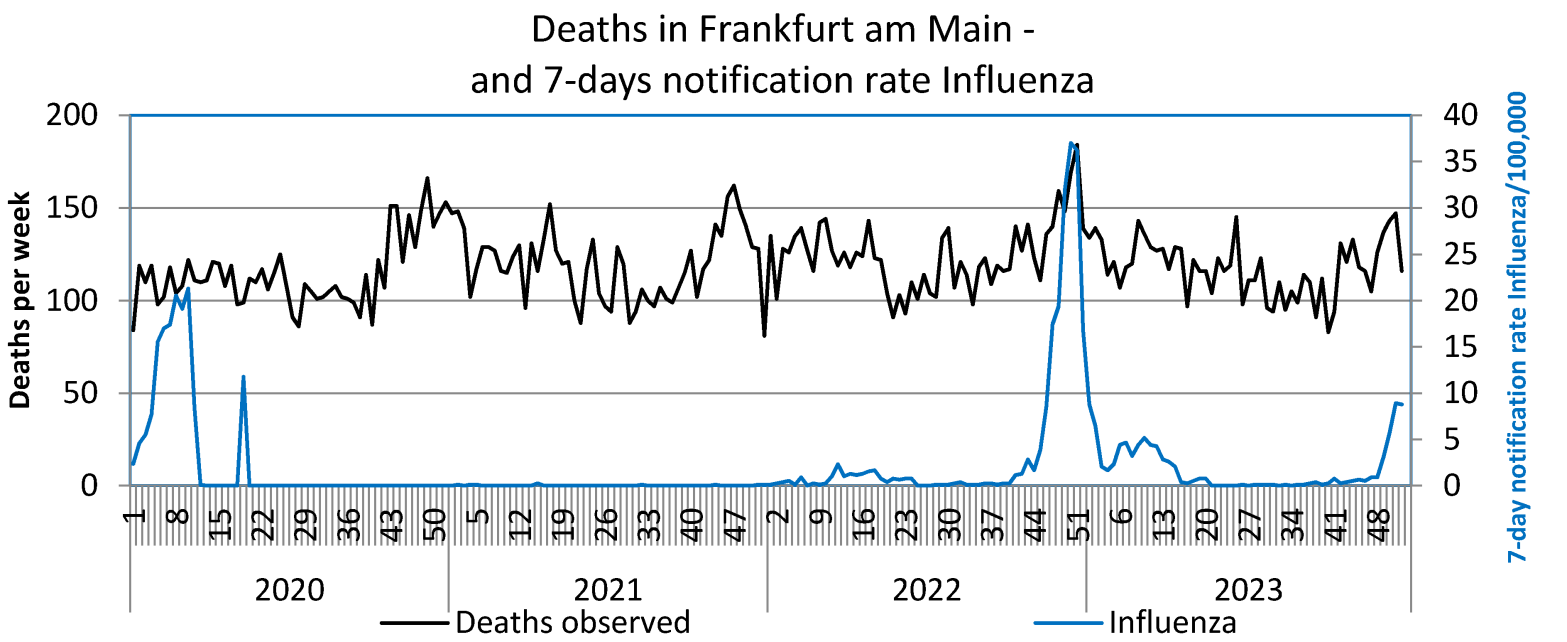
Weekly deaths (unadjusted) and 7-day notification rates influenza/100,000 in Frankfurt am Main, Germany

**Figure 4:**
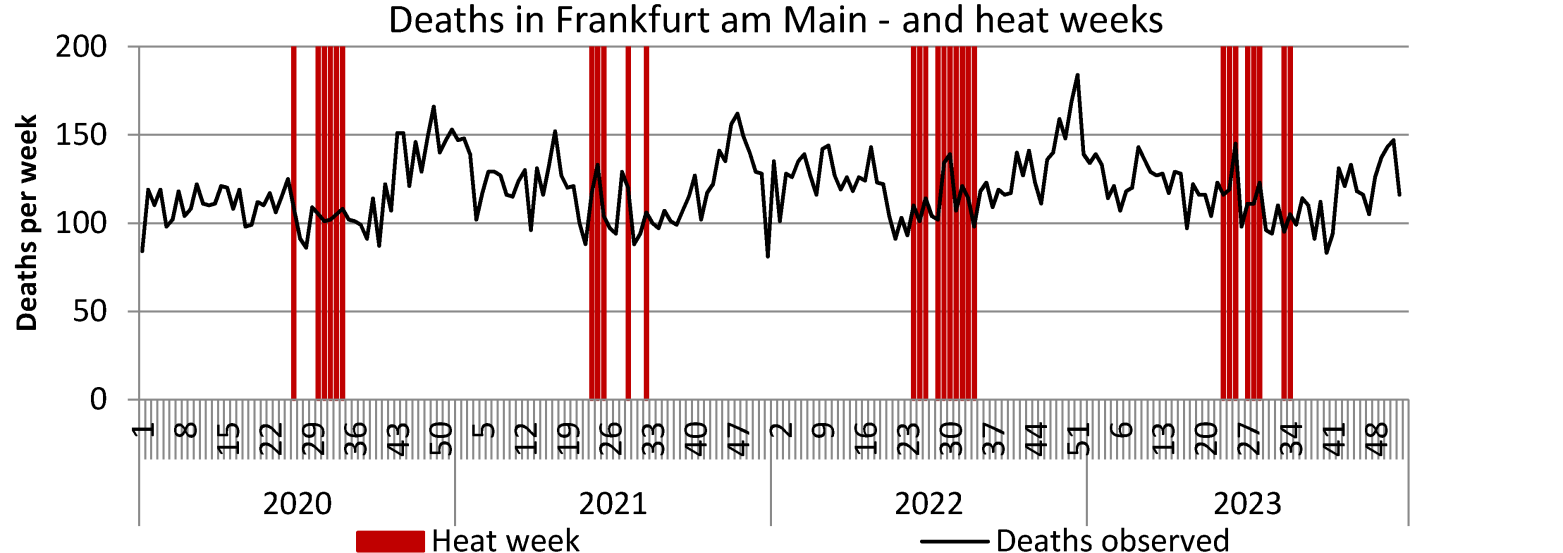
Weekly deaths (unadjusted) and heat weeks in Frankfurt am Main, Germany (heat weak: week with weekly average temperature ≥20°C)

SARS-CoV-2 reports in the first waves up to the end of 2021 reached a maximum of 350/week per 100,000, in the 5^th^ wave (week 52/2021–21/2022) reporting rates up to 2,500/week per 100,000 were achieved, in the 6^th^ wave (week 22–33/2022) peaks of up to 1,000 reports and in the 7^th^ wave (week 34–46/2022) up to approx. 700/week per 100,000 were documented. These extremely high reporting rates in 2022 were neither associated with a noticeable increase in observed deaths nor with the SMR. However, an increase in observed deaths and the SMR towards the end of 2022, i.e. after the three further waves of the pandemic, is striking. The peak in deaths in week 51/2022 coincides with the peak in influenza reports (Figure 3 (Fig. 3)). The renewed increase in deaths per week at the end of 2023 is also accompanied by an increase in influenza reports (Figure 3 (Fig. 3)). Some peaks in weekly deaths in the summer months 2021–2023 are coincident with heat weeks (Figure 4 (Fig. 4)). 

Table 2 (Tab. 2) shows SARS-CoV-2 waves 1 to 7 according to the dominant pathogen, duration of the waves, notifications of SARS-CoV-2 observed and expected deaths and excess mortality as a difference and as a standardized mortality ratio with the corresponding confidence intervals. While a negative excess mortality (SMR 0.917, 95% CI 0.866–0.969; deaths –110.5) was recognizable in the first wave and a positive excess mortality (SMR 1.106, 95% CI 1.066–1.147, excess deaths +274) in the second wave, no significant deviations in the SMR were detected in any of the other waves with the other SARS-CoV-2 variants. Overall, excess mortality in the 65 weeks of the first 4 waves of the pandemic amounted to SMR 1.023, correspondending to 189 excess deaths.

In the three subsequent waves with the dominant omicron variant of the SARS-CoV-2 virus, the SMR – despite very high reported SARS-CoV-2 case numbers – was 0.988, which corresponds to a calculated negative excess mortality of 66 cases. In the last 6 weeks of 2022, i.e. after the 7^th^ wave of the pandemic, there was a SMR 1.250 (95% CI 1.170–1.330) and 188 excess deaths during increased influenza reports. 

The excess mortality during the heat weeks in the summer months 2020–2023 is depicted in Table 3 (Tab. 3). In the summer months of June–August 2020 to 2023, 7 (2020), 4 (2021), 10 (2022) and 8 (2023) heat weeks were observed in Frankfurt am Main [20]. In the heat weeks of 2020, a negative excess mortality (SMR 0.937; 95% CI 0.864–1.001), –42 deaths) was observed, positive excess mortality was found: +38 cases (2021), +20 cases (2022) and +50 cases (2023). The SMRs did not increase significantly. 

**Figure 5:**
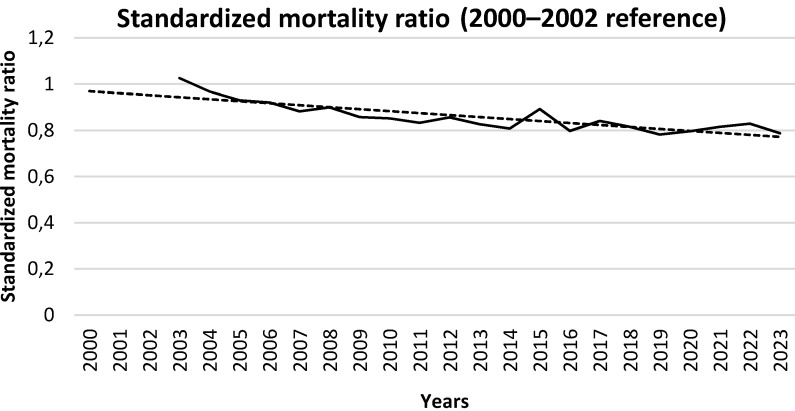
Age trend adjusted standardized mortality ratios – reference 2000–2002 – in Frankfurt am Main, Germany, 2000–2023

Between 2000 and 2023, the population in Frankfurt am Main increased from 613,886 to 767,434 inhabitants, which corresponds to an increase of 25%. The annual number of deaths per 100,000 decreased from 1,006 to 802 (minus 20%). The standardized mortality ratios using 2000–2002 as a reference and adjusted for age and population trends decreased from 1.025 to 0.788 between 2003 and 2023 (Table 4 (Tab. 4) and Figure 5 (Fig. 5)). The decrease was interrupted by 2015 with a summer heat wave, and by 2022 with the influenza wave, whereas during the COVID-19 pandemic there was no discernible deviation from the long-term trend.

Weekly influenza reports remained below 10/100,000 until 2008, rose to 86/100,000 in the influenza pandemic year 2009 and were consistently above 100/100,000 from 2017 onwards, with the exception of 2021, when only very few cases were reported (1.2/100,000). In 2018, the maximum reporting rate for influenza was 313/100,000; another high reporting rate of 199/100,000 was recorded in 2022. In contrast, the SARS-CoV-2 reporting rates were 1–2 orders of magnitude higher (Table 4 (Tab. 4)).

**Figure 6:**
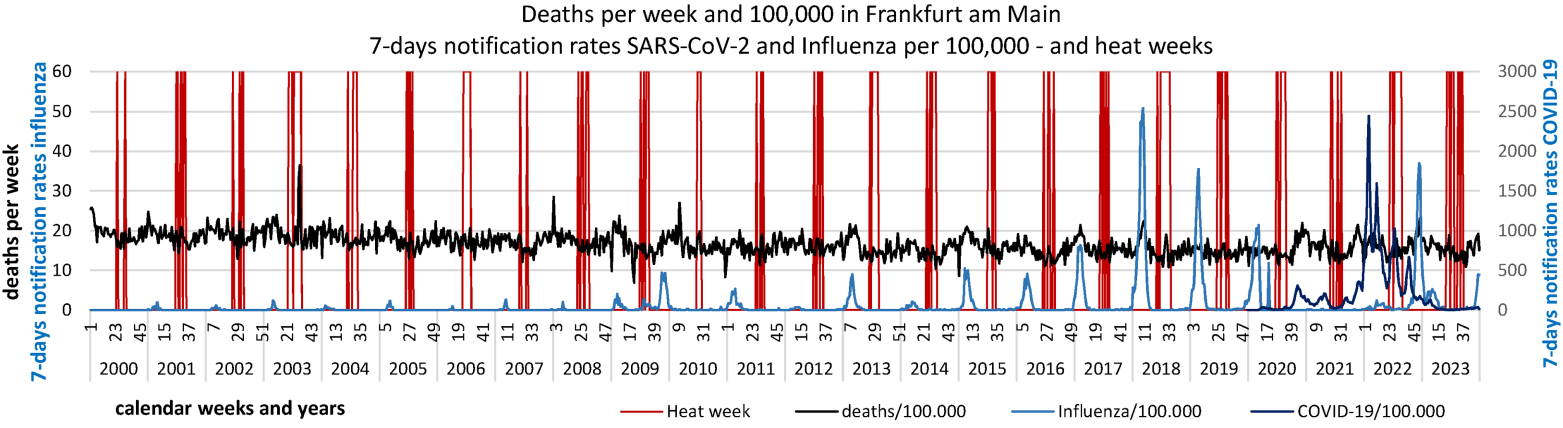
Weekly deaths, COVID-19 and influenza notifications/100,000 and heat weeks in Frankfurt am Main, Germany, 2000–2023

The presentation of observed deaths per 100,000 by calendar week for the years 2000 to 2023 shows the typical annual pattern of mortality with higher death counts in winter and lower death counts in the summer months. Looking at the pandemic years, there is only a small “winter peak” overall in 2020, followed by higher “winter peaks” in 2021 to 2023. In addition, very high weekly death rates can also be seen in individual heat weeks in 2003, 2018 and 2023, with the absolute highest weekly death rate during the enormous heat wave in August 2003 (Figure 6 (Fig. 6)).

## Discussion

**Method:** in principle, excess mortality calculations can be strongly influenced by the choice of method. This applies not only to the choice of age groups but also to the reference periods chosen to calculate the standardized mortality ratio SMR [5]. There are no standardized, generally accepted specifications either for the age groups to be used for age standardization or for the reference periods [5], [6]. Therefore, Ioannidis et al. [10] suggested publishing not just one, but possibly several calculations in order to present this alternative in a transparent manner. Against this background, we compare the calculations based on 8 age groups (see Attachment 1) with those from our work based on 5 age groups as an example, and also provide a further reference period of 3 years for both methods in addition to the reference period of 4 years chosen for our work. It is apparent, that in the Frankfurt population the further differentiation of the higher age groups results in higher values for mortality (differences and SMRs), while the consideration of a shorter reference period of 3 years results in slightly lower mortality rates. However, this may well be different in populations with a different age structure and population development. We decided to to continue using the 5 age groups and 4 reference years, as this is also established [4] and we were thus able to provide a direct follow-up to our earlier publication [12]. 

It is also worth discussing whether reference periods dating back years are appropriate, e.g. the years 2016–2019 as a reference for the year 2023. Shorter intervals would usually be preferred. However, when considering the pandemic, only the pre-pandemic years made sense as reference years, not the inclusion of years in which the pandemic situation already existed. Against this background, we decided – as did other authors [10] – to use only the pre-pandemic years for our analysis.

**Results:** In summary, the update of the assessment of mortality adjusted for age and population trends in the years 2020–2023 in Frankfurt am Main shows a significant excess mortality in 2022, followed by a significant negative excess mortality in 2023. In the years 2020 and 2021 however, no significant deviation in excess mortality was found.

Regarding the impact of SARS-CoV-2 on excess mortality, an excess mortality of 10.6% with an increase of 274 deaths was detected during the 19 weeks of the 2^nd^ wave at the end of 2020 before the introduction of the vaccination. In wave 2, there was also a noticeably high number of deaths in care facilities for the elderly in Frankfurt [21]. During the first four waves of the pandemic as a whole (wild type, alpha and delta variants), there was an excess mortality ratio of 2.3% and 189 excess deaths. 

The SMRs in 2020 and 2021 in Frankfurt am Main were lower than the numbers calculated for Germany by Ioannidis et al. using a similar adjustment for the age and population trend, with 5 age groups and the reference years 2017–2019 (+2.4%) [10]. They were also well below the excess mortality ratios calculated for Germany using a different method (2020: 0.41%; 2021: 3.43%) [22]. Kuhbandner and Reitzner [22] described a sudden increase in excess mortality in Germany from April 2021 and proposed possible vaccination (side) effects as the cause. Based on our data this cannot be confirmed, because a comparable increase was not seen in Frankfurt am Main. 

In the three subsequent pandemic waves in 2022, with the dominant omicron variant of the SARS-CoV-2 virus, the SMR – despite very high reported SARS-CoV-2 case numbers – was 0.988, which corresponds to a calculated negative excess mortality of 66 cases. Therefore, SARS-CoV-2 infections during pandemic waves 5–7 cannot explain the excess mortality in 2022.

Our evaluations thus underline that, in agreement with studies from many countries, especially the so-called non-vulnerable countries [10], there was a high excess mortality rate in Frankfurt in the 2^nd^ wave only before the introduction of the vaccination, but during the 7 waves of the SARS-CoV-2 pandemic in Frankfurt am Main, there was no significant excess mortality overall. The reasons for this cannot be deduced from the data available to us. Prevention measures may be discussed. Legally binding non-pharmaceutical measures, such as lockdown, contact restrictions (ban on major events, restrictions on visits, e.g., in care facilities for the elderly; school closures or distance learning, restrictions on visiting stores, restaurants, etc.), obligatory testing or protective measures such as the requirement to wear masks in many settings, even outdoors, had been ordered. However, there is no reliable data on the real implementation of these measures, such as the correct wearing of masks, etc. Furthermore, in Sweden, for example, the age-standardized mortality rate – after the first wave with many deaths, especially in elderly care facilities – was lower than in Germany, although Sweden refrained from school closures, lockdowns and mandatory masks [10]. 

Heat also has an impact on mortality in the population, and heat events might have influenced the excess mortality, especially in 2022. The calculated excess mortality in Frankfurt am Main amounted to a total of 66 deaths during the heat weeks of 2020–2023, 20 of these in summer 2022. Since the SARS-CoV-2 reports outside the pandemic waves were very low in the summer months of 2020 and 2021 and the high SARS-CoV-2 reports in wave 6 in summer 2022 were not associated with any excess mortality, it may be plausibly assumed that the above-mentioned excess deaths during the heat weeks represent increased heat-related mortality. 

During recent decades, the fundamental influence of heatwaves on mortality among the population in Frankfurt has been evident in mortality peaks during summer weeks, with the increase in mortality during the major heatwave in August 2003 being particularly impressive. Such an extreme increase in deaths did not occur in any other heatwave in Frankfurt, possibly due to the education and training measures introduced afterwards [20]. The evaluations of the Federal Statistical Office [23] and the European mortality statistics [24] also consistently show mortality peaks in summer weeks for Germany. Against this background, the effects of summer heat on the mortality of the population should not be underestimated. However, they cannot explain the excess mortality in Frankfurt am Main in the year 2022. 

Closer examination showed that the excess mortality in 2022 was due to very high excess mortality during the last 6 weeks (SMR 1.250; 95CI 1.170–1.330), i.e., the period after the 7^th^ wave of the pandemic. Numerous influenza cases were reported during these weeks. The excess mortality in these 6 weeks amounted to 188 deaths, i.e., a higher excess mortality than was recorded cumulatively over all pandemic waves (n=123). The excess mortality during that influenza wave in the last weeks of 2022 was 31/week, and thus twice as high as during the second wave of the SARS-CoV-2 pandemic when it was 14/week.

In Germany as a whole, deaths also increased at the end of 2022, from an annual average of under 20,000 per week to over 28,000 in week 51/2022, coinciding with an increase in reports of influenza cases to 60,023 in week 50/2022 [15], [25], [26]. Moreover, data from the Federal Ministry of Health’s infection radar confirms an extremely high incidence of respiratory infections in Germany at the end of 2022 [27]. With 3,217, the number of doctor visits recorded in the sentinel practices in the week from 12–18 December 2022 was higher than at any time during the entire SARS-CoV-2 pandemic; the second-highest rate was 2,123 respiratory infections in the week from 14–20 March 2022, 33% lower. Moreover, hospitalizations (38.1/100,000) due to severe respiratory diseases in the week from 12–18/12/2022 were also much higher than the next highest rate during the pandemic (25.2/100,000 in the week from 22–28/2021). Intensive-care bed occupancy, which had never exceeded the 90% mark during the entire pandemic, also peaked at 90.1% on December 21^st^, 2022. At the same time, there was no significant increase in reported COVID-19 deaths in Germany [27]. Influenza waves were also observed in other countries at the end of 2022, with an apparently strong influence on mortality [11].

It is therefore plausible to conclude that the excess mortality in 2022 in Frankfurt am Main can be attributed to a wave of influenza at the end of the year. This led to a higher excess mortality than in all pandemic waves, even in the second wave before the introduction of vaccination but under intensive protective measures. It is worth noting that some of the protective measures were still in place during that influenza wave, such as hygiene measures in schools, and FFP-2 masks had been made compulsory on public transport only a few weeks earlier. However, as mentioned above, no reliable data on the actual implementation of the measures is known here either. In addition, considering the frequent waves of influenza that occurred in previous years and the vaccinations, a certain basic or cross-immunity against the influenza viruses circulating at the end of 2022 could also be assumed, a finding that cannot be transferred to the situation of the completely unvaccinated population during the second COVID-19 wave.

## Strengths and limitations

Our small-scale study only examines a small population, which limits its transferability to other regions. However, the small-scale approach allows parallel consideration of other potential influencing factors in addition to SARS-CoV-2 notifications, such as heat waves and influenza waves.

A strength of our work is the long observation period over 4 years (total) and the consideration of further potential influencing factors in addition to SARS-CoV-2 on mortality in Frankfurt. 

A different age standardization and choice of reference period may lead to different results; this has been impressively demonstrated by various authors. However, the method we chose is well established [4].

When defining the waves according to calendar weeks, we were only able to use the definition of the RKI [16] up to wave 6, not for the definition of wave 7, as no information has yet been published by the Robert Koch-Institute. Different definitions could lead to different results.

The increase in SARS-CoV-2 notifications over the years was influenced by the increasing availability of tests and the increasing instances in which testing was obligatory, so that although the duration of the pandemic waves was easily comparable over time, the number of weekly SARS-CoV-2 notifications was not. And a comparison with the influenza notifications was also not possible either, as there were no testing obligations for asymptomatic persons as with SARS-CoV-2 and the tests were done according to medical indication only.

We can only show associations of mortality with SARS-CoV-2 or influenza reports or heat waves. A statement on causality is not possible based on our data.

## Conclusion

During the SARS-CoV-2 pandemic, excess mortality occurred in Frankfurt am Main only in the second wave at the end of 2020 before vaccination was introduced (+10.6%, +274 deaths); in all subsequent waves, no significant excess mortality was recorded. Encompassing all pandemic-waves excess mortality was 0.9% (+123 deaths). Overall, there was a non-significant negative excess mortality in Frankfurt am Main in 2020 and 2021 and a significant negative excess mortality in 2023. In 2022, however, a significant excess mortality of +2.9% (+185 excess deaths) was observed, which could not be attributed to SARS-CoV-2 but to a short, intense wave of influenza in the last 6 weeks at the end of that year, which had also led to a significant increase in mortality throughout Germany. This wave of influenza was associated with an excess mortality rate of 25% (+188 deaths) in Frankfurt am Main. It was thus higher than in any wave of the SARS-CoV-2 pandemic in Frankfurt am Main, as well as higher than in all pandemic waves together. This remarkable fact should be taken into account when dealing with evaluating the pandemic, a process which is increasingly being called for in Germany but is still pending. 

## Notes

### Competing interests

The authors declare that they have no competing interests.

### Authors’ ORCID

- Ursel Heudorf: 0000-0002-0050-8272
- Bernd Kowall: 0000-0003-4163-1696

### Funding

None

## Supplementary Material

Mortality in Frankfurt am Main, Germany, 2020–2023, unadjusted and adjusted for age trend and population development using 5 resp. 8 age groups, and different reference periods

## Figures and Tables

**Table 1:**
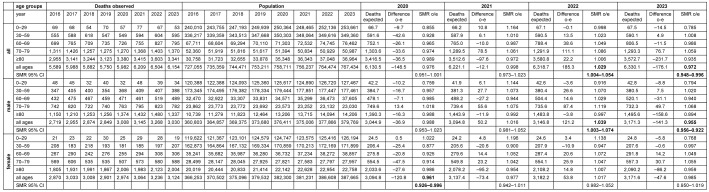
Mortality in Frankfurt am Main, Germany, 2020–2023, adjusted for age trend and population development

**Table 2:**
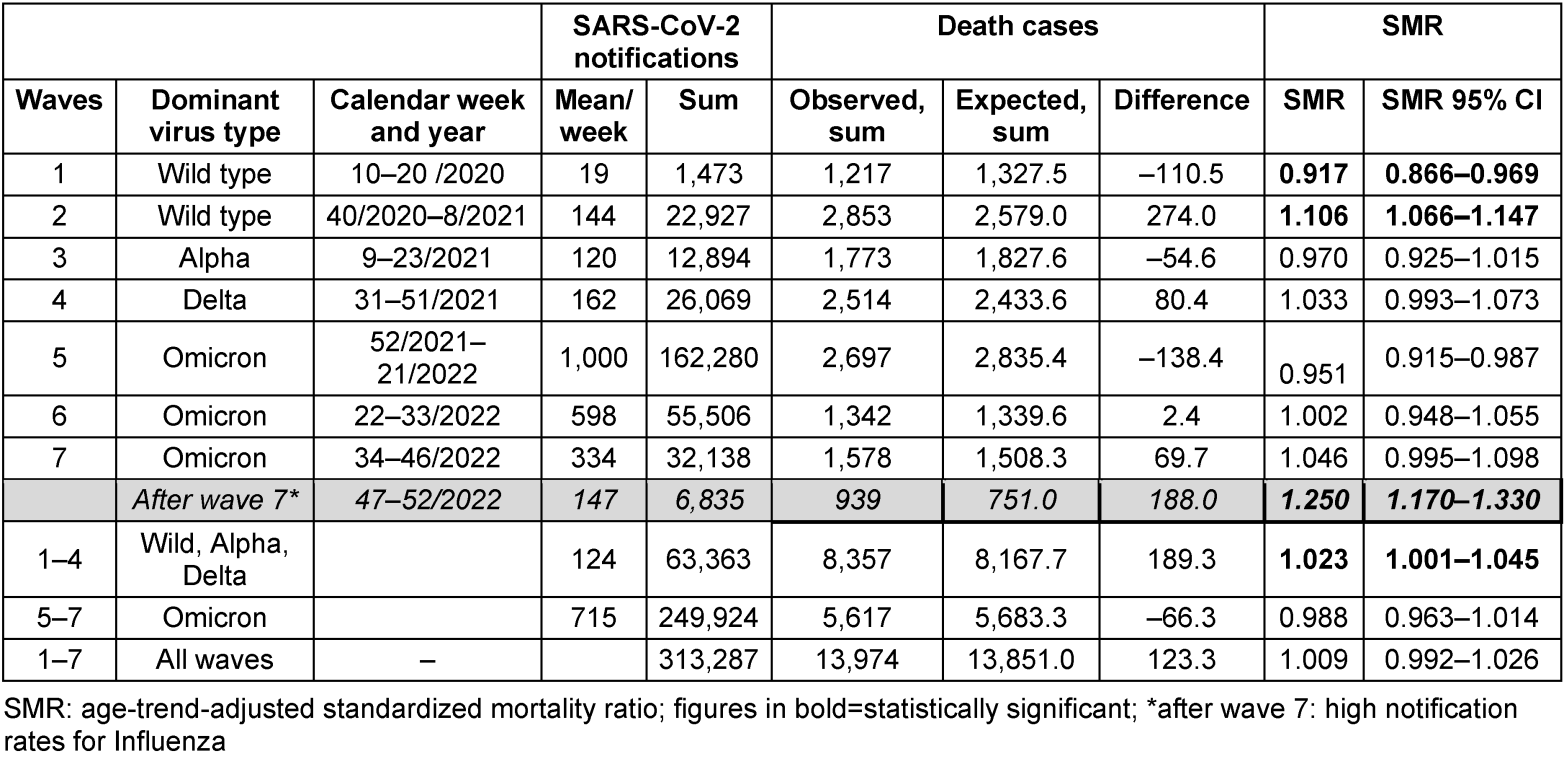
SARS-CoV-2 Pandemic waves 1–7 by dominant pathogen, time and duration, notified SARS-CoV-2 cases, observed and expected deaths and standardized mortality ratios (SMR) in Frankfurt am Main, Germany

**Table 3:**
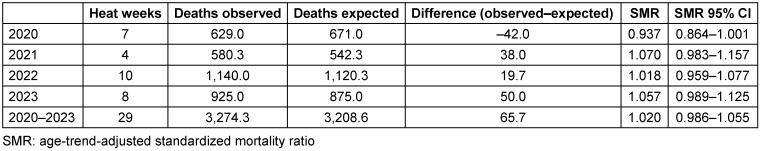
Heat weeks and excess mortality (differences and SMR) in Frankfurt am Main, Germany, June–August 2020–2023

**Table 4:**
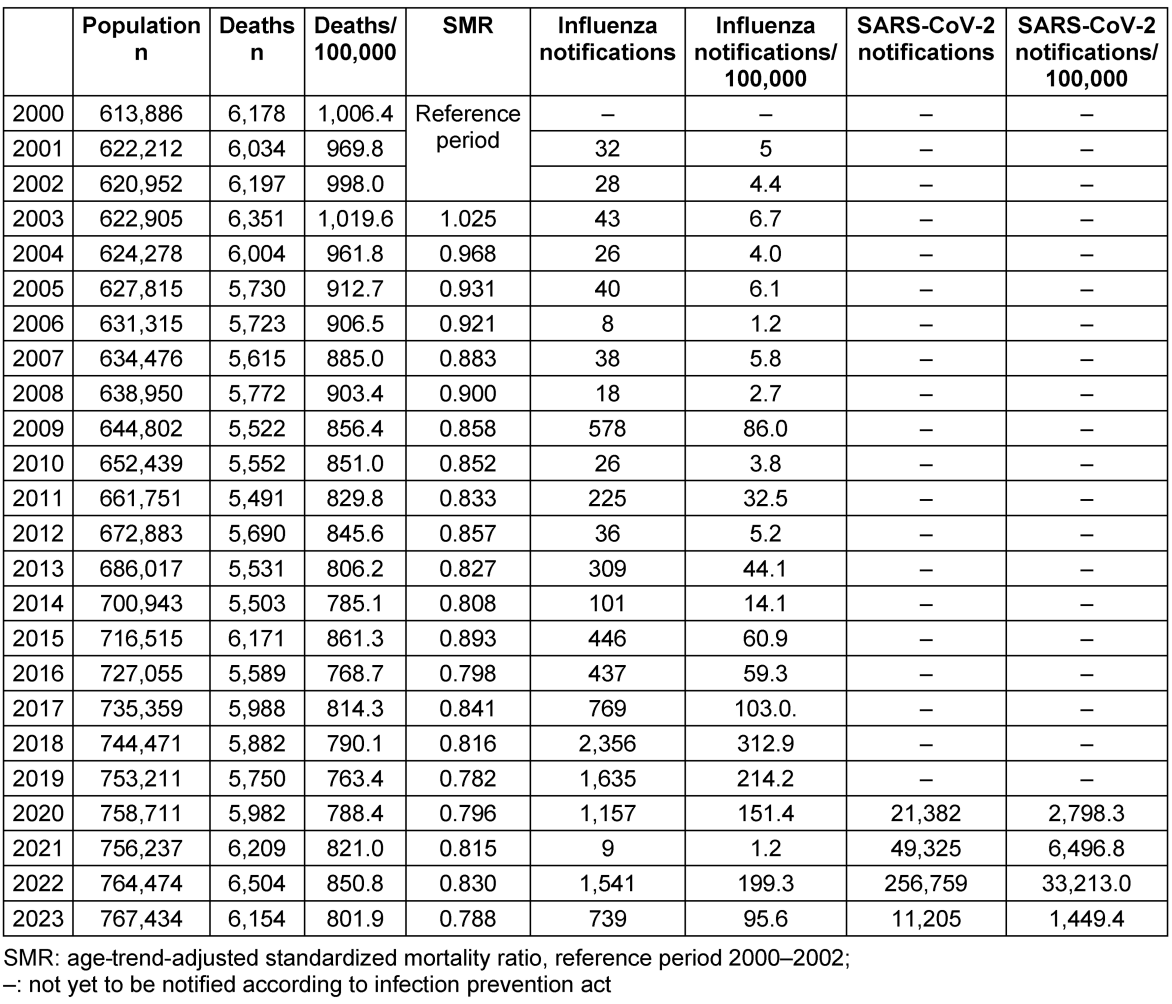
Population development, deaths, standardized mortality ratios, influenza, and SARS-CoV-2 notifications per year as well as per 100,000 in Frankfurt am Main, Germany, 2000–2023
