# Supplementary material for: Mortality in Frankfurt am Main, Germany, 2020–2023: higher excess mortality during an influenza wave in 2022 than during all COVID-19 waves altogether
Source: GMS Hyg Infect Control. 2025 Mar 4;20:Doc04. doi: 10.3205/dgkh000533 (PMC12059809; doi:10.3205/dgkh000533)
Supplement: Mortality in Frankfurt am Main, Germany, 2020–2023, unadjusted and adjusted for age trend and population development using 5 resp. 8 age groups, and different reference periods [file HIC-20-04-s-001.pdf]

Attachment 1

Mortality in Frankfurt am Main, Germany, 2020–2023, unadjusted and adjusted for age trend and population development using 5 resp. 8 age groups, and different reference periods

		Deaths observed								Population								2020			2021			2022			2023		
	**Age (years)**	**2016**	**2017**	**2018**	**2019**	**2020**	**2021**	**2022**	**2023**	**2016**	**2017**	**2018**	**2019**	**2020**	**2021**	**2022**	**2023**	**Deaths expected**	**Difference (o–e)**	**SMR**	**Deaths expected**	**Difference (o–e)**	**SMR**	**Deaths expected**	**Difference (o–e)**	**SMR**	**Deaths expected**	**Difference (o–e)**	**SMR**
**No adjustment for age**																													
Reference period 2016–2019	all	5,589	5,988	5,882	5,750	5,982	6,209	6,504	6,154	727,055	735,359	744,471	753,211	758,711	756,237	764,474	767,434	5,949.2	32.8	1.006	5,929.8	279.2	**1.047**	5,994.4	509.6	**1.085**	6,017.7	136.3	1.023
95% CI																				(0.980–1.031)			**(1.021–1.073)**			**(1.059–1.111)**			(0.997–1.048)
Reference period 2017–2019																			–6.2	0.999		240.3	**1.040**		470.3	**1.078**		96.9	1.016
95% CI																				(0.974–1.024)			**(1.014–1.066)**			**(1.052–1.104)**			(0.001–1.041)
**Adjustment 5 age groups**	0–29	69	68	54	70	57	77	67	53	240,010	243,755	247,193	249,939	250,364	248,465	252,136	253,661	66.7	–9.7	0.855	66.2	10.8	1.164	67.1	–0.1	0.998	67.5	–14.5	0.785
	30–59	555	588	618	547	549	594	604	595	336,217	339,359	343,513	347,668	350,303	348,064	349,616	349,360	591.6	–42.6	0.928	587.9	6.1	1.010	590.5	13.5	1.023	590.1	4.9	1.008
	60–69	699	765	709	735	726	755	827	795	67,711	68,604	69,294	70,110	71,303	72,532	74,745	76,462	752.1	–26.1	0.965	765.0	–10.0	0.987	788.4	38.6	1.049	806.5	–11.5	0.986
	70–79	1,311	1,426	1,257	1,275	1,270	1,368	1,403	1,370	52,360	51,919	51,816	51,617	51,394	50,834	50,929	50,987	1,303.6	–33.6	0.974	1,289.5	78.5	1.061	1,291.9	111.1	1.086	1,293.3	76.7	1.059
	≥80	2,955	3,141	3,244	3,123	3,380	3,415	3,603	3,341	30,758	31,723	32,655	33,878	35,348	36,343	37,048	36,964	3,416.5	–36.5	0.989	3,512.6	–97.6	0.972	3,580.8	22.2	1.006	3,572.7	–231.7	0.935
Reference period 2016–2019	all	5,589	5,988	5,882	5,750	5,982	6,209	6,504	6,154	727,055	735,359	744,471	753,211	758,711	756,237	764,474	767,434	6,130.5	–148.5	0.976	6,221.1	–12.1	0.998	6,318.7	185.3	**1.029**	6,330.1	–176.1	**0.972**
95% CI																				(0.951–1.001)			(0.973–1.023)			**(1.001–1.051)**			**(0.948–0.996)**
Reference period 2017–2019	all																		–168.9	**0.976**		–32.8	0.995		164.3	**1.026**		–197.2	**0.969**
95% CI																				**(0.948–0.997)**			(0.970–1.019)			**(1.001–1.051)**			**(0.945–0.993)**
**Adjustment 8 age groups**	0–29	69	68	54	70	57	77	67	53	240,010	243,755	247,193	249,939	250,364	248,465	252,136	253,661	66.7	–9.7	0.855	66.2	10.8	1.164	67.1	–0.1	0.998	67.5	–14.5	0.785
	30–59	555	588	618	547	549	594	604	595	336,217	339,359	343,513	347,668	350,303	348,064	349,616	349,360	591.6	–42.6	0.928	587.9	6.1	1.010	590.5	13.5	1.023	590.1	4.9	1.008
	60–64	284	314	293	308	337	327	369	341	35,762	36,421	37,070	37,912	39,142	40,236	41,663	42,871	318.9	18.1	1.057	327.8	–0.8	0.997	339.5	29.5	1.087	349.3	–8.3	0.976
	65–69	415	451	416	427	389	428	458	454	31,949	32,183	32,224	32,199	32,161	32,297	33,082	33,591	427.5	–38.5	0.910	429.3	–1.3	0.997	439.8	18.2	1.041	446.5	7.5	1.017
	70–74	546	561	500	521	537	575	618	592	26,522	26,045	26,305	26,627	27,542	28,372	28,855	28,791	555.7	–18.7	0.966	572.4	2.6	1.005	582.2	35.8	1.062	580.9	11.1	1.019
	75–79	765	865	757	754	733	793	785	778	25,838	25,875	25,511	24,991	23,852	22,462	22,074	22,196	732.8	0.2	1.000	690.1	102.9	1.149	678.1	106.9	1.158	681.9	96.1	1.141
	80–84	894	948	974	975	1,025	1,109	1,126	1,043	15,708	16,677	17,771	18,826	19,722	20,217	20,282	19,794	1,086.5	–61.5	0.943	1,113.8	–4.8	0.996	1,117.3	8.7	1.008	1,090.5	–47.5	0.956
	≥80	2,061	2,193	2,270	2,148	2,355	2,306	2,477	2,298	15,050	15,046	14,884	15,052	15,626	16,126	16,766	17,170	2,257.6	97.4	1.043	2,329.8	–23.8	0.990	2,422.3	54.7	**1.023**	2,480.7	–182.7	0.926
Reference period 2016–2019	all	5,589	5,988	5,882	5,750	5,982	6,209	6,504	6,154	727,055	735,359	744,471	753,211	758,711	756,237	764,474	767,434	6,037.2	–55.2	0.991	6,117.2	91.8	1.015	6,236.8	267.2	**1.043**	6,287.4	–133.4	**0.979**
95% CI																				(0.966–1.016)			(0.990–1.040)			**1.017–1.068**			**0.954–1.003**
Reference period 2017–2019	all																		–96.0	0.984		50.0	1.008		224.3	**1.036**		–177.7	**0.972**
95% CI																				(0.959–1.009)			(0.983–1.003)			**1.011–1.061**			**0.948–0.996**

SMR: standardized mortality ratio; o: observed; e: expected

Attachment to: Heudorf U, Kowall B. Mortality in Frankfurt am Main, Germany, 2020-2023: higher excess mortality during an influenza wave in 2022 than during all COVID-19 waves altogether. GMS Hyg Infect Control. 2024;19:Doc04. DOI: 10.3205/dgkh000533
